# TSP‐1 is downregulated and inversely correlates with miR‐449c expression in Cushing's disease

**DOI:** 10.1111/jcmm.14297

**Published:** 2019-04-23

**Authors:** Jie Ren, Changwei Gu, Yong Yang, Jun Xue, Yuhao Sun, Fangfang Jian, Dongjiang Chen, Liuguan Bian, Qingfang Sun

**Affiliations:** ^1^ Department of Neurosurgery, Ruijin Hospital Shanghai Jiaotong University School of Medicine Shanghai P.R. China; ^2^ Department of Neurosurgery, Ruijin Hospital, Luwan Branch Shanghai Jiaotong University School of Medicine Shanghai P.R. China; ^3^ Department of Neurosurgery Guangdong General Hospital, Guangdong Academy of Medical Sciences Guangzhou China; ^4^ Department of Obstetrics and Gynecology, Ruijin Hospital Shanghai Jiaotong University School of Medicine Shanghai P.R. China; ^5^ Department of Neurosurgery, McKnight Brain Institute University of Florida Gainesville Florida

**Keywords:** ACTH‐secreting pituitary adenomas, Cushing's disease, lncTHBS1, miR‐449c, thrombospondin‐1

## Abstract

The pathogenesis of Cushing's disease, which is caused by pituitary corticotroph adenoma, remains to be studied. Secreted angioinhibitory factor thrombospondin‐1 (TSP‐1) is an adhesive glycoprotein that mediates cell‐to‐cell and cell‐to‐matrix interactions and is associated with platelet aggregation, angiogenesis and tumorigenesis. We have found that the expression of TSP‐1 is significantly lower in human pituitary corticotroph tumours compared with normal adenohypophysis. This study aims to elucidate the role of TSP‐1 in regulating the tumour function of pituitary adenomas. Forced overexpression of TSP‐1 in a murine AtT20 pituitary corticotroph tumour cell line decreased corticotroph precursor hormone proopiomelanocortin (POMC) transcription and adrenocorticotropic hormone (ACTH) secretion. Functional studies showed that TSP‐1 overexpression in pituitary adenoma cells suppressed proliferation, migration and invasion. We have demonstrated that TSP‐1 is a direct target of miR‐449c. Further study showed that miR‐449c activity enhanced tumorigenesis by directly inhibiting TSP‐1 expression. Low expression of lncTHBS1, along with low expression of TSP‐1, was associated with the high expression of miR‐449c in Cushing's disease patients. Furthermore, RNA‐immunoprecipitation associates miR‐449c with lncTHBS1 suggesting that lncTHBS1 might be a negative regulator of miR‐449c. Taken together, this study has demonstrated that lncTHBS1 might function as competing endogenous RNA for miR‐449c, which could suppress the development of Cushing's disease.

## 1 INTRODUCTION

Cushing's disease is a consequence of the chronic hypercortisolism associated with the oversecretion of the adrenocorticotropic hormone (ACTH) and is the most common cause of corticotrophin‐dependent Cushing's syndrome, accounting for up to 80% of cases.^1^, ^2^, ^3^, ^4^ Secreting pituitary adenomas that cause Cushing's disease are usually benign slow‐growing tumours originating from pituitary corticotroph cells.^5^ The secretion of excessive ACTH promotes the adrenal glands to produce elevated levels of cortisol, which then induces endogenous hypercortisolism.^6^ Approximately one‐ to two‐third(s) of corticotroph adenomas possess somatic mutations in the 14‐3‐3 protein binding motif of ubiquitin specific peptidase 8 (USP8), a deubiquitinating enzyme gene.^7^, ^8^ Mutations in USP8 may be associated with some of the clinical features of Cushing's disease such as tumour aggressiveness and ACTH hypersecretion.^9^, ^10^ However, the symptoms of Cushing's disease are similar to other conditions and often remain unrecognized, especially in individuals diagnosed with diabetes, hypertension or depression.^11^ In fact, the mortality rates in patients with untreated Cushing's disease are reported to be up to nine times higher those of the general population.^12^, ^13^ Pituitary adenomectomy is generally performed in the first‐line treatment of Cushing's disease, however, pituitary adenomas often recur after remission.^2^ Therefore, more effective therapeutic and diagnostic approaches are needed to improve the survival prospects of patients with Cushing's disease.

Thrombospondin‐1 (TSP‐1) is a matricellular protein first found in human plasma and widely expressed in diverse tissue.^14^ TSP‐1 is a multi‐domain glycoprotein that is encoded by the *THBS1* gene and involved in cell‐to‐cell and cell‐to‐matrix interactions, in particular, it is associated with platelet aggregation, angiogenesis and tumorigenesis.^15^, ^16^, ^17^ Using RNA sequencing (RNA‐Seq), the transcriptome of 13 cases of CD and five normal human pituitaries (NHPs) were analysed in our previous study.^18^ Marked downregulation of the TSP‐1 encoding gene was identified in Cushing's disease. TSP‐1 has been demonstrated to have a complicated role in human cancer and to exert stimulatory and inhibitory effects in different types of tumours. TSP‐1 is known as an inhibitor of proliferation in endothelial cells^19^ and induces apoptosis,^20^ and suppresses the cell cycle.^21^ TSP‐1 is under‐expressed in various tumours such as colorectal cancer,^22^ clear cell renal carcinoma.^23^ The upregulation of TSP‐1 can suppress tumour growth in stroma^24^ and oesophageal squamous cell carcinoma.^25^ Moreover, it was reported that activated somatostatin receptor subtype2 (sst2) and bone morphogenetic protein 4 (BMP4) can also suppress the growth of solid tumours, such as in pancreatic and cervical cancers, via the induction of TSP‐1.^26^, ^27^ Interestingly, use of an sst2 analog and BMP4 can also inhibit corticotroph tumour cells and ACTH secretion as well.^28^, ^29^ In addition, through the activation of TGF‐beta, TSP‐1 may inhibit angiogenesis and tumour growth in multiple cancers.^30^ It is also known that the downregulation of the TGF beta signalling pathway and activation of the TGF pathway may decrease the secretion of ACTH and tumour cell proliferation in pituitary corticotrophinomas.^31^, ^32^ However, TSP‐1 may, in contrast, be involved in the promotion of tumorigenesis in various cancers such as gastric cancer and human melanoma.^33^, ^34^ TSP‐1 induced by TGFB1 is reported to promote the migration of oral squamous cell carcinoma and stimulate the expression of matrix metalloproteinases (MMPs) through integrin signalling.^35^ However, the decreased expression of TSP‐1 and its cause in Cushing's disease remains to be elucidated. TSP‐1 is proposed to influence the vascular endothelial growth factor (VEGF) pathway by binding to a high‐affinity receptor CD47 and disrupting its association with VEGF receptor 2, which in turn downregulates the pro‐angiogenic signals downstream of VEGF.^36^ Ki67, VEGF and matrix metalloproteinase‐9 (MMP9) are among the markers normally used to identify the biochemical characteristics of Cushing's disease.^37^, ^38^, ^39^, ^40^, ^41^

MicroRNAs (miRNAs) are short non‐coding RNA molecules with 22‐24 nucleotides, which can affect the stability and translation of mRNAs through binding to targeted mRNA. Several miRNAs, such as miR‐26a and miR‐449a, also play an important role in the regulation of ACTH‐secreting pituitary adenomas.^42^, ^43^ It has been hypothesized that glucocorticoids may induce the expression of miRNAs in the pituitary. The 3′untranslated region (UTR) of TSP‐1 is a potential target of miR‐449c.

Long non‐coding RNAs (lncRNAs) are a group of miRNAs that could function as a miRNA sponge, often referred to as competing endogenous RNA (ceRNA), regulating the expression pattern and biological characteristics of miRNA. Other studies implicate lncRNAs in the regulation of pituitary adenomas and other cancers.^44^, ^45^, ^46^ An elevated level of lncRNA H19 expression was found in invasive pituitary adenoma cells.^44^ LncRNA CCAT2 has recently been found to be significantly upregulated in pituitary adenomas tissues.^45^ Loss‐ and gain‐of‐function assays showed that CCAT2 positively regulated pituitary adenoma cell proliferation, migration and invasion, and interacted with PTTG1 to promote its stability.

To investigate dysregulated lncRNAs and miRNAs in Cushing's disease we selected miR‐449c and lncTHBS1 and compared their expression in ACTH‐secreting adenoma and normal pituitary tissue. Northern blot analysis, luciferase assay and RNA‐immunoprecipitation assay were used to determine the reciprocal relationship between miR‐449c and lncTHBS1. We also investigated the expression of TSP‐1 in ACTH‐secreting adenoma and its effects on the expression of POMC, VEGF, Ki67 and MMP9.

## 2 MATERIALS AND METHODS

### 2.1 Human tissue samples and cell culture

Human pituitary tumour samples were obtained by transsphenoidal surgery from 33 patients with Cushing's disease. Normal human pituitaries (NHP) (n = 7) were obtained from fresh autopsy specimens. This study was approved by the Ruijin Hospital Ethics Committees and written informed consent was obtained from all patients. HEK‐293T and the mouse AtT20 pituitary corticotroph tumour cell line were obtained from the American Tissue Type Collection (ATCC, Manassas, VA, USA). Cells were maintained in Dulbecco's modified Eagle's medium (DMEM, GIBCO, Carlsbad, CA, USA) with 10% foetal bovine serum (FBS), 2 mmol/L L‐glutamine and 100 μg/mL penicillin/streptomycin in a humidified incubator with 5% CO_2_ at 37°C.

### 2.2 Plasmid construction

TSP‐1 and negative controls were obtained from GenePharma (Shanghai, China). The full‐length 3′‐UTR of TSP1 was inserted into a pGL3‐Control luciferase vector (Promega, Madison, WI, USA) following the manufacturer's instructions. All primers used in the study can be found in Table 1. The pRenilla‐TK vector was used as an internal control for the dual‐luciferase assay. Cells were grown to 60%‐70% confluence then seeded into six‐well plates 24 hours before infection. The cells in each well were transfected with 1 mL of culture medium containing 10 μL of lentivirus (1×10^9^ TU/ml) and incubated for 72 hours, the infection rate was observed using a fluorescence microscope(Olympus, Center Valley, PA, USA). Western blotting was used to confirm effective transfection.

**Table 1:** Primers used in the study

H‐TPS‐1‐S	GACAGCATCCGCAAAGTGACT
H‐TPS‐1‐A	CATTGGAGCAGGGCATGATGG
hsa‐miR‐449c‐RT	GTCGTATCCAGTGCAGGGTCCGAGGTGCACTGGATACGACACAGCCG
U6‐RT	GTCGTATCCAGTGCAGGGTCCGAGGTGCACTGGATACGACAAAATATGG
hsa‐miR‐449c	ACACTCCAGCTGGGTAACAGTCTACAGCCA
LncTHBS1‐S	CTGAAATGAGATTGCCTGAGCTG
LncTHBS1‐A	GCTGCACCTTTCACGTCTAGTTT
U6‐S	CTCGCTTCGGCAGCACA
U6‐A	AACGCTTCACGAATTTGCGT
H‐GAPDH‐S	GGAAGCTTGTCATCAATGGAAATC
H‐GAPDH‐A	TGATGACCCTTTTGGCTCCC
Mus‐TPS‐1‐S	CCTGCCAGGGAAGCAACAA
Mus‐TPS‐1‐A	ACAGTCTATGTAGAGTTGAGCCC
Mus‐miR‐449c‐S	TGCGCAGGCAGTGCATTGCTA
Mus‐miR‐449c‐A	CCAGTGCAGGGTCCGAGGTATT
Mus‐Mmp2‐S	CTGCCACTGTCCCAGGAAG
Mus‐Mmp2‐A	CTCGCGGCAAGTCTTCAGAG
Mus‐Mmp7‐S	CTTACCTCGGATCGTAGTGGA
Mus‐Mmp7‐A	CCCCAACTAACCCTCTTGAAGT
Mus‐Mmp9‐S	GCAGAGGCATACTTGTACCG
Mus‐Mmp9‐A	TGATGTTATGATGGTCCCACTTG
Mus‐Pomc‐S	ATGCCGAGATTCTGCTACAGT
Mus‐Pomc‐A	TCCAGCGAGAGGTCGAGTTT
Mus‐Gapdh‐S	AGGTCGGTGTGAACGGATTT
Mus‐Gapdh‐A	GGGGTCGTTGATGGCAACA

### 2.3 Immunohistochemistry

Tissues were fixed in 4% paraformaldehyde for at least 24 hours, dehydrated and paraffin embedded. Sections were cut at 5 μm from representative formalin‐fixed, paraffin wax‐embedded blocks and floated onto positively charged slides (SuperFrost Plus; Menzel‐Glaser, Portsmouth, NH, USA). The slides were dewaxed in xylene and rehydrated through graded ethanol. Histological sections were incubated with primary goat antibodies against Ki67, VEGF, MMP9 and TSP‐1 (Santa Cruz Biotechnology, Dallas, TX, USA) overnight at 4°C. Sections were washed in PBS and incubated with FITC‐conjugated (1:200, Proteintech, Chicago, IL, USA) goat anti‐rabbit IgG for 1 hour. Sections were then counterstained with DAPI for 10 minutes. The images were acquired with a microscope (Olympus). The staining intensity and distribution was graded, and the immunoreactive score was calculated as intensity of the staining multiplied by the percentage of positive distribution which has been described in our previous study.^18^

### 2.4 RNA preparation and qRT‐PCR

Total RNA was extracted from cell cultures and tumour tissue samples using TRIzol reagent (Life Technologies, Inc, Gaithersburg, MD, USA) and reverse‐transcribed using HiScript Reverse Transcriptase (GeneCopoeia, Rockville, MD, USA). The RNA purity was measured using a NanoDrop® Spectrophotometer (NanoDrop Technologies). The cDNA was synthesized from 1 g deoxyribonuclease‐treated RNA with an oligo‐dT primer using a Revert Aid First Strand cDNA Synthesis kit (Promega) following the manufacturer's protocol. Real‐time PCR was performed using SYBR‐green PCR Master Mix (TaKaRa, Beijing, China) in a FastReal‐time PCR 7500 System (Applied Biosystems, Foster City, CA, USA). The PCR was performed with the following parameters: 50°C for 2 minutes, followed by 40 cycles of 95°C for 15 seconds and 60°C for 1 minute. The primers used in the study can be found in Table 1. Relative expression of mRNAs, lncTHBS1and miR‐449c was calculated using the 2^−ΔΔCT^ method and normalized to GAPDH and U6, respectively.

### 2.5 Western blotting assays

After cells were lysed in RIPA buffer (Beyotime, Shanghai, China) containing 1 mmol/L PMSF (Beyotime) and 0.1% protease inhibitor cocktail (Roche, Basel, Switzerland) for 30 minutes at 4℃, protein concentrations were determined (BCA Protein Assay Kit, Fisher Scientific Waltham. MA, USA). Protein samples (30 μg) were then separated by 10% SDS‐PAGE and transferred onto polyvinylidene fluoride membranes (Millipore, Billerica, MA,USA). Membranes were blocked in 5% non‐fat dried milk and then incubated at 4℃ overnight with primary TSP‐1 (1:1000, sc‐59886, Santa Cruz), GAPDH (1:2000, 10494‐1‐AP, Proteintech), MMP9 (1:1000, 10375‐2‐AP, Proteintech) and VEGF (1:1000, MAB293, R&D Systems) followed by incubating with horseradish peroxidase‐conjugated secondary antibody for 2 hours at room temperature. Protein bands were visualized using an enhanced chemiluminescence Amersham^TM^ ECL Plus Western blotting detection system (GE Healthcare, Amersham, UK).GAPDH was used as internal loading control.

### 2.6 Cell viability assays and colony formation test

To determine cell viability, AtT20 cells were seeded into 96‐well plates (2000 cells/well) and incubated for 24 hours until they were attached. Cell viability was determined using a Cell Counting Kit‐8 (Dojindo Laboratories, Kumamoto, Japan) following the manufacturer's instructions. Colony formation was determined by culturing AtT20 cells in 6‐well plates (10^3^ cells/well) in DMEM culture medium containing 10% FBS, which was replaced every 4‐5 days. After 21 days, colonies were fixed for 20 minutes in 4% paraformaldehyde in PBS containing 4% sucrose and then stained with 0.005% crystal violet for 30 minutes. After washing in PBS, colonies were counted using an inverted microscope.

### 2.7 Cell apoptosis analysis (Annexin V assay)

Apoptosis was assessed by flow cytometry using an AnnexinV‐FITC apoptosis detection kit (Beyotime, Shanghai, China). After harvesting, cells were washed twice with cold PBS and then stained for 10 minutes at room temperature in 1 mL Annexin V binding buffer with 10 μL of PI solution (100 µg/ml; Sigma, Israel) and 5 μL of Annexin V‐FITC (2.5 µg/ml, BD Pharmingen). The number of apoptotic cells was analysed on a FACS Caliber flow cytometer (Becton Dickinson, Franklin Lakes, NJ, USA).

### 2.8 Cell migration and invasion assays

To assess migration, cells (1×10^5 ^cells/ml) were seeded into each chamber of culture‐insert microdishes (Ibidi GmbH, Planegg, Germany) in 100 μL of culture medium. Following 24 hours incubation at 37°C and serum starving for another 6 hours, inserts were removed and 1 ml of DMEM with 2% FBS was added to each dish. Images were obtained after the removal of the insert and 48 hours later. Cells that migrated into the gaps were counted using Image J software (NIH, Bethesda, MA, USA) to estimate the rate of migration.

For the cell invasion assay, 8 mm pore Transwell inserts (BD Biosciences, Heidelberg, Germany) were coated with 75 μL Geltrex Matrix (Geltrex, Life Technologies, Frankfurt, Germany) and used as upper chambers in a 24‐well plate. Cells (1×10^5 ^cells/ml) in serum‐free medium were added to the upper chambers and incubated at 37°C for 48 hours. Cells that migrated through the Geltrex Matrix to the bottom membrane of the insert were fixed with 10% formalin, stained with hematoxylin solution and counted at 200× magnification.

### 2.9 ELISA assay

ACTH ELISA kit (EK‐001‐21, Phoenix Pharmaceuticals) was used to detect the level of ACTH. Corticosterone ELISA kit (ALPCO, 55‐CORMS‐E01) was used to detect the level of corticosterone. Briefly, the culture media of AtT20 cells was collected from each well after overexpressing miR‐449c or TSP‐1 for 48 hours for further measurement. Then, the kit was used following the manufacturer's instruction. Each experimental group was analysed six times and the experiments were repeated independently three times.

### 2.10 In vivo xenograft tumour model

Six‐week‐old nu/nu male mice were inoculated subcutaneously with empty vector or TSP‐1 overexpressing stable transfectant AtT20 cells (5×10^5^ cells per mouse).The mice were killed 21 days after tumour cell implantation. The tumours (n = 3 per group) were harvested and photographed. The length (L) and width (W) of the tumours were measured with a digital calliper. The tumour volume (V) was calculated using the formula V = (L × W^2)/2). Tissues samples and blood samples were collected on the day of killing and embedded for histological examination and ELISA assay. Ki‐67 expression in tumour sections was evaluated by immunohistochemical staining. All of the animal procedures were conducted in accordance with the Guide for The Care and Use of Laboratory Animals published by the National Institutes of Health.

### 2.11 Northern blot analysis

Total RNA (20 μg) was prepared using TRIzol reagents (Life Technologies) was denatured with formaldehyde and loaded on to agarose gel containing 1.2% formaldehyde. After electrophoresis, the RNA was transferred to a nylon membrane and fixed with a UV crosslinker. The membrane was probed with a digoxigenin‐labelled lncTHBS1 oligonucleotide probe with the following sequence: 5′‐tgcacggggtgggatcgcggcaccgcactctggta‐3′ at a concentration of 10 pmol/L, overnight at 42°C. Blots were processed using a Brightstar Detection Kit (Ambion Inc, Austin, TX, USA). A GADPH probe (5′‐ggtgctaagcagttggtggtgcaggaggcattgct‐3′) was used as an internal control.

### 2.12 RNA‐immunoprecipitation assays

RNA‐IP was conducted in HEK‐293T cells (ATCC, Manassas, VA, USA) 48 hours after transfection with miR‐449c mimics or miR‐NC (miRNA negative control), using a Magna RIP™ RNA‐binding protein immunoprecipitation kit (Millipore, Billerica, MA, USA) following manufacturer's instructions. Cells (1×10^7^) were lysed in complete RNA lysis buffer, then cell lysates were incubated with RIP immunoprecipitation buffer containing magnetic beads conjugated with human anti‐Argonaute2 (AGO2) antibody (Millipore, Billerica, MA, USA) or negative control mouse IgG (Millipore, Billerica, MA, USA). Samples were incubated with Proteinase K and then immunoprecipitated RNA was isolated. Extracted RNAs were analysed by RT‐PCR or qRT‐PCR to identify the presence of lncTHBS1.

### 2.13 Dual‐luciferase assay

The lncTHBS1 promoter was amplified from human genomic DNA and then subcloned into a pGL3 vector (Promega) to construct a wild‐type lncTHBS1 promoter and a promoter with mutated binding sites (Gene Copoeia, Guangzhou, China). After transfection, 3×10^4^cells were seeded in 48‐well plates and incubated for 24 hours. The pTSP‐1‐luciferase plasmid (100 ng, Clontech), or control‐luciferase plasmid, was transfected with 1 ng of pRL‐TK Renilla plasmid (Promega) into cells using Lipofectamine 2000 (Invitrogen). Luciferase and Renilla activity was measured using a Dual‐Luciferase Reporter Assay Kit (Promega) according to the manufacturer's instructions. Firefly luciferase activity was normalized to Renilla luciferase activity for each individual analysis.

### 2.14 Statistical analysis

Comparisons were analysed using a two‐tail unpaired Student's *t*test or one‐way ANOVA. The Mann‐Whitney *U* test was used for continuous variables. All statistical analysis was performed using SPSS version 16.0. The association between clinicopathological parameters and TSP‐1 expression were evaluated by *χ* ^2^ test. Values are presented as mean ± standard deviation unless otherwise stated and *P < *0.05 was considered to be statistically significant.

## 3 RESULTS

### 3.1 TSP‐1 is downregulated in human pituitary corticotroph adenoma

**Figure 1:**
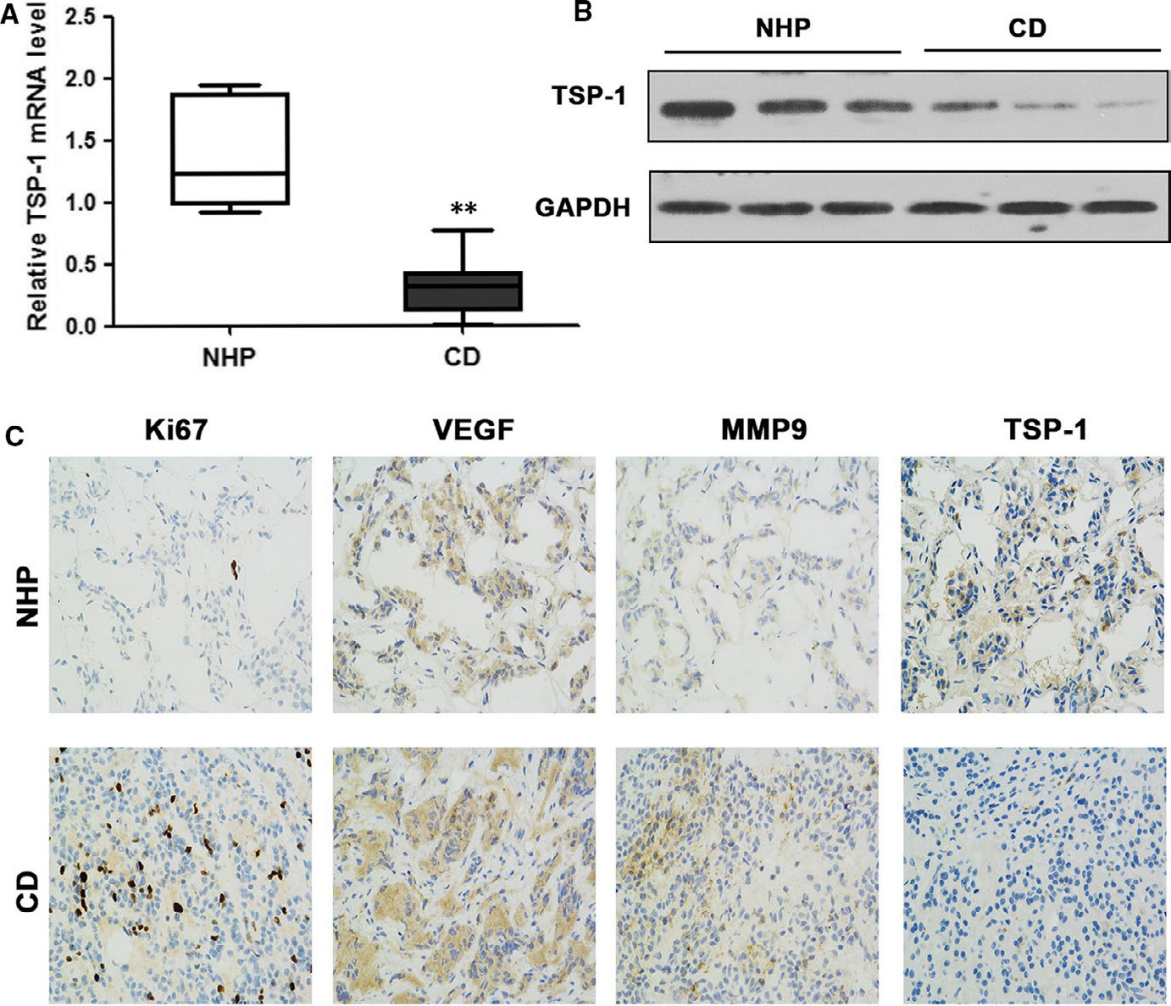
Relative TSP‐1 expression in pituitary corticotrophs. A, The expression of TSP‐1 mRNA in Cushing's disease (CD, n = 33) and normal human pituitary (NHP, n = 7) tissue. B, Western blotting of TSP‐1 protein levels in normal pituitary and CD tissue samples. C, Immunohistochemical staining for Ki67, VEGF, MMP9 and TSP‐1 in representative normal pituitary and corticotroph adenoma (magnification, ×200). Representations of at least three biological replicates are presented (mean ± SEM; **P < 0.01)

The relative expression of TSP‐1 was determined in normal pituitary tissue (n = 7) and in the pituitary tissue of patients with Cushing's disease (n = 33). The expression of TSP‐1 mRNA and protein levels was found to be significantly lower in Cushing's disease than in normal pituitary tissue (*P* < 0.01) (Figure 1A,B). Protein levels of Ki67, VEGF, MMP9 and TSP‐1 were also assessed by immunohistochemical staining and counterstained with DAPI (Figure 1C). The presence of Ki67 was more intense in the nuclei of tissue samples from patients with Cushing's disease, which indicates increased cell proliferation. The location of VEGF and MMP9 appeared to be more cytoplasmic and they were upregulated. The staining of TSP‐1 was clearly less intense in the pituitary tissue of patients with Cushing's disease compared with normal pituitary tissue. These results confirm that TSP‐1 is downregulated in Cushing's disease. The clinicopathological features of these patients with CD were collected. Based on the Knosp/Hardy classification, we classified a total of 33 cases as invasive (n = 9) and non‐invasive (n = 24). Moreover, microadenoma (n = 28) and macroadenoma (n = 5) are also classified based on a diameter less or more than 10 mm, respectively. The analysis of TSP‐1 expression and association with these clinicopathologies is shown in Table 2.

**Table 2:** TSP‐1 expression and clinicopathological features in patients with Cushing's disease

Parameters	TSP‐1		Total	*P*‐value
	Low (%)	High (%)		
Age (years old)				0.151
＜45	13 (39.4)	6 (18.2)	19	
≥45	11 (33.3)	3 (9.1)	14	
Gender				0.718
Female	14 (42.4）	9 (27.3)	23	
Male	6 (18.2）	4 (12.1)	10	
Invasiveness				0.301
Yes	7 (21.2)	2 (6.1)	9	
No	14 (42.4)	10 (30.3)	24	
Tumour size (cm)				0.045
Microadenoma	25(75.8)	3 (9.1)	28	
Macroadenoma	5 (15.2)	0 (0.0)	5	
Plasma ACTH (pg/ml)	251.27 ± 83.29	194.31 ± 106.32	33	0.625

### 3.2 TSP‐1 suppresses tumorigenicity in vitro and in vivo

**Figure 2:**
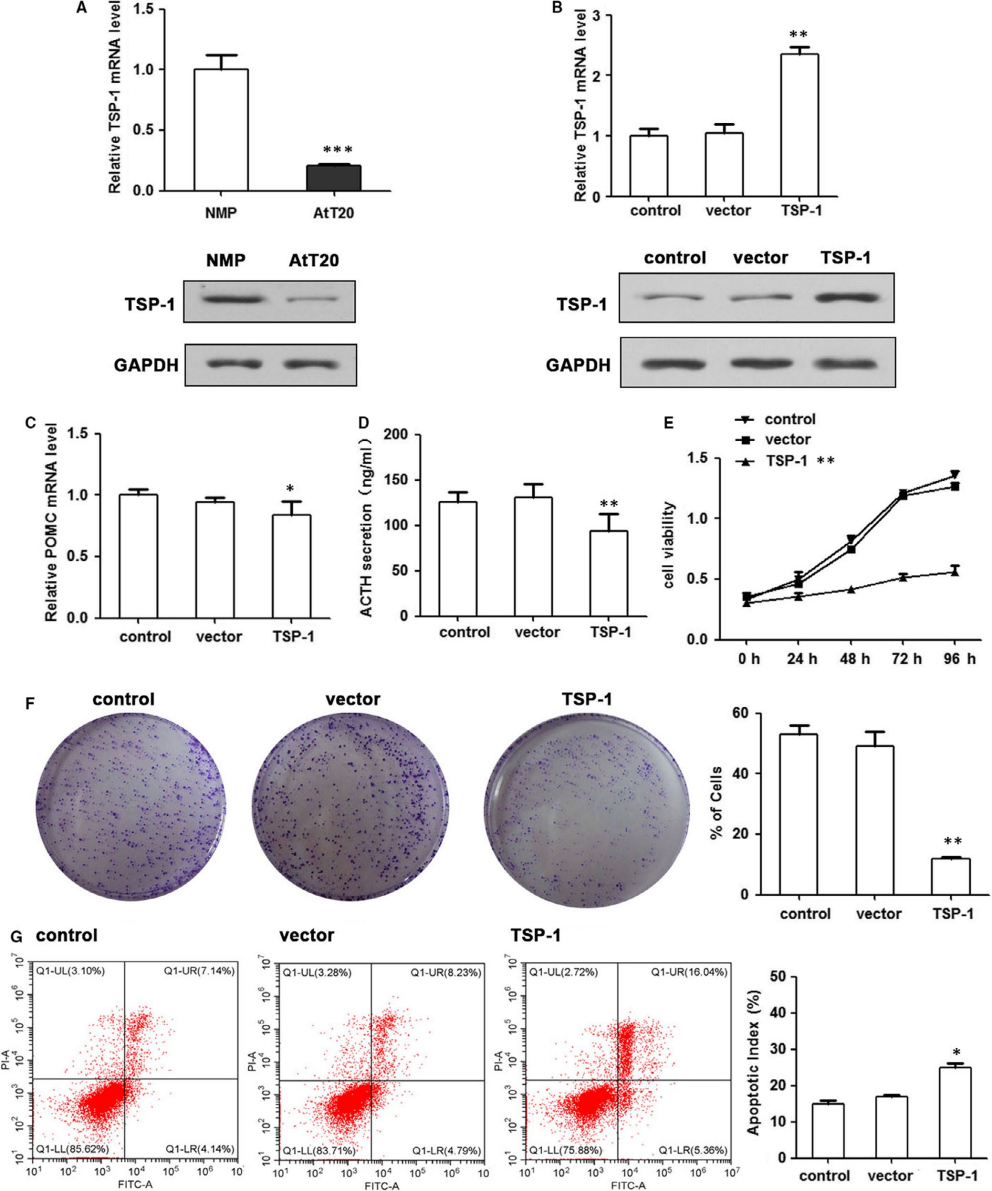
TSP‐1 decreases the proliferation and clonogenic ability of AtT20 cells in vitro. Overexpression of TSP‐1 levels through the transfection of pcDNA‐TSP‐1 in AtT20. A, The relative expression of TSP‐1 was measured by qRT‐PCR and WB. B, qRT‐PCR and WB assay confirmed increased expression of TSP‐1 in stably transfected AtT20 cells. C, TSP‐1 effects on POMC expression was detected by qRT‐PCR (n = 3; *P < 0.05, **P < 0.01, ***P < 0.001 vs control). D, TSP‐1 effects on adrenocorticotropic hormone (ACTH) secretion was detected by ELISA assay. E, MTT assay showed that overexpression TSP‐1 decreased cell growth rates. F, Colony formation was assessed under a microscope. (n = 3, *P < 0.05, **P < 0.01). G, Cell apoptosis was determined by flow cytometry. Mean ± SEM; *P < 0.05, **P < 0.01, ***P < 0.001

**Figure 3:**
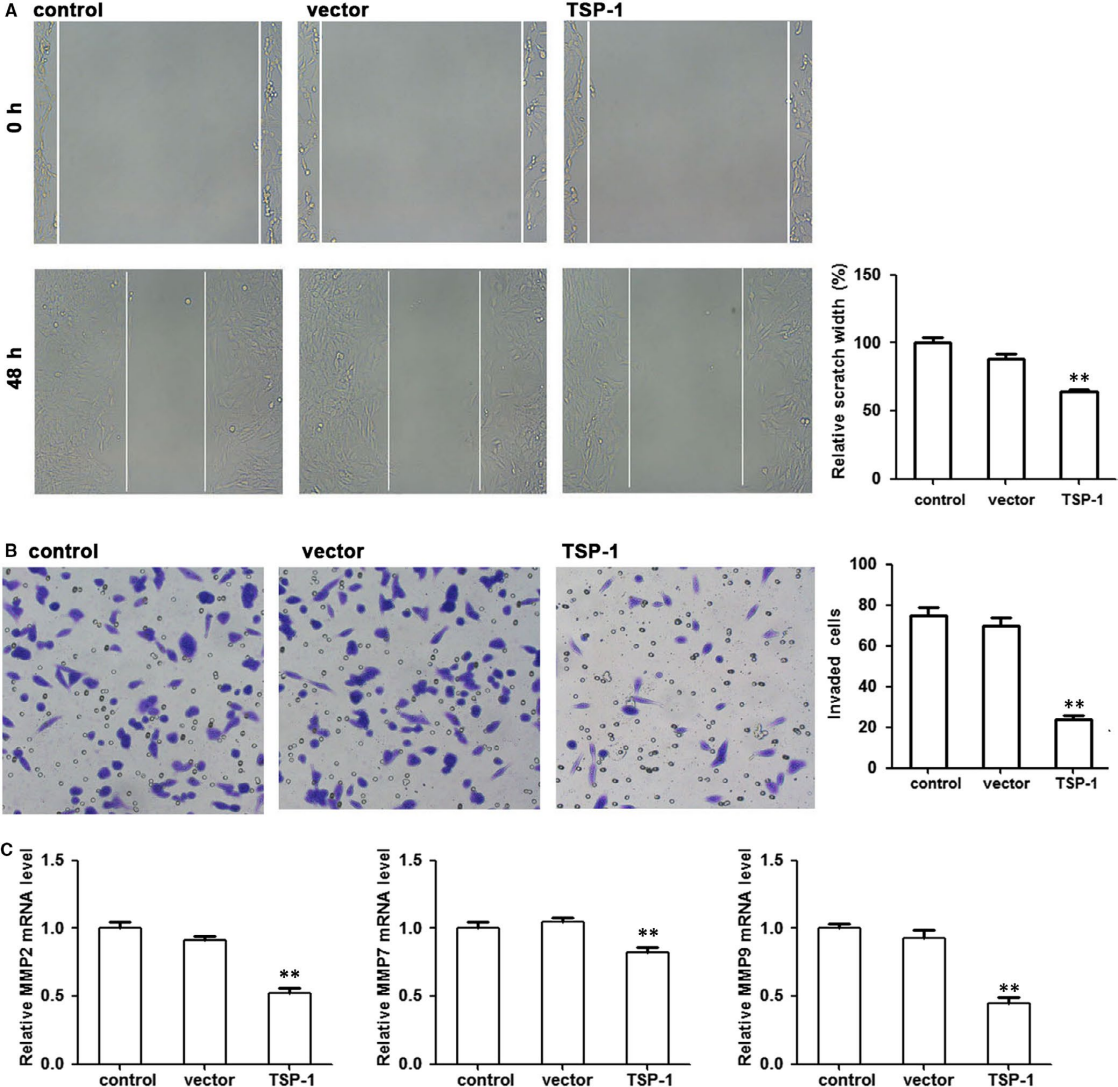
TSP‐1 inhibits AtT20 cell migration. A, Representative images of the wound healing assay in the TSP‐1‐transfected or vector‐transfected AtT20 cells at 48 h. B, Transwell invasion assay was employed to assess the invasive potential of overexpressing TSP‐1 in AtT20 cells. C, Relative expression of MMP2, MMP7 and MMP9 by qRTPCR. **P < 0.01

To further investigate the influence of TSP‐1 on cell proliferation, migration and invasion we transfected a murine AtT20 pituitary corticotroph tumour cell line with a TSP‐1 expression vector (pcDNA‐TSP‐1). The relative expression of TSP‐1 measured by qRT‐PCR and WB was significantly lower in untransfected AtT20 cells but levels were elevated in cells transfected with pcDNA‐TSP‐1 (Figure 2A,B). We found that overexpression of TSP‐1 decreased corticotroph precursor hormone POMC transcription and ACTH secretion in AtT20 cells and significantly decreased cell viability and colony formation (Figure 2C‐F). Moreover, flow cytometric analysis indicated that TSP‐1 overexpression increased the ratio of apoptosis (Figure 2G). TSP‐1 overexpression was also found to inhibit AtT20 cell migration in a wound healing assay conducted over 48 hours (Figure 3A) and Transwell invasion was also reduced by the overexpression of TSP‐1 in AtT20 cells (Figure 3B). In addition, the relative expression of the matrix metalloproteinases MMP‐2, MMP‐7 and MMP‐9 was reduced by the overexpression of TSP‐1 indicating that TSP1 expression could prevent the degradation of extracellular matrix proteins (Figure 3C). Overall our results indicate that the overexpression of TSP‐1 decreases the proliferation and clonogenic ability of AtT20 cells in vitro and suppresses migration and invasion.

**Figure 4:**
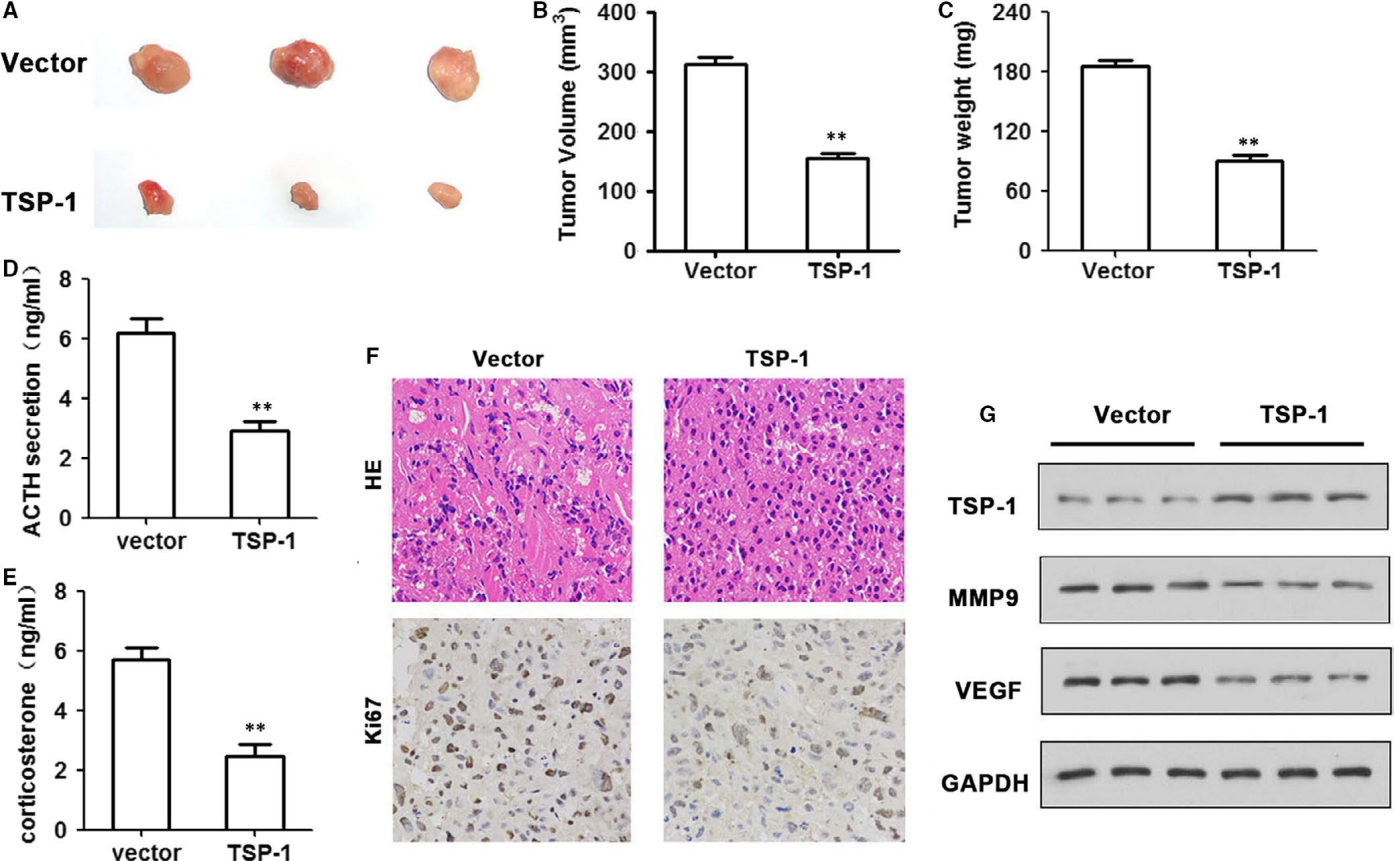
TSP‐1 inhibits tumour growth in vivo. A, TSP‐1‐overexpressed AtT20 cells were injected subcutaneously in the nude mice. Mice were killed 21 d later for evaluation. Gross morphology of tumour is shown. B, TSP‐1 upregulation decreased the volume of tumours compared with the control group (n = 6 per group). (D‐E) ACTH (D) and corticosterone (E) levels derived from mice harbouring stable TSP‐1 overexpressing cells compared with controls by ELISA assay. F, Hematoxylin‐eosin staining of samples and immunoreactivity to Ki67 as observed by fluorescence microscopy (magnification ×400). G, The protein expression of TSP‐1, VEGF and MMP9 was measured by Western blotting. GAPDH was used as an internal control. Representations of at least three biological replicates are presented (mean ± SEM; **P < 0.01)

To determine the influence of TSP‐1 overexpression on tumorigenicity, tumour growth was assessed in nude mice that were subcutaneously injected in the right flank with either control AtT20 cells or AtT20 cells overexpressing TSP‐1. Tumour volume and weight were the lowest in TSP‐1 overexpression mice (Figure 4A‐C). We also detected the expression levels of ACTH and corticosterone in blood samples of nude mice (Figure 4D‐E). HE and Ki67 expression were detected in tumours by IHC (Figure 4F). TSP‐1, MMP‐9, VEGF expression were detected by Western Blot (Figure 4G). Ki67, MMP‐9 and VEGF levels are reduced. Taken together, these results indicate that overexpressing TSP‐1 decreases tumour growth in nude mice.

### 3.3 TSP‐1 inversely correlates with miR‐449c expression in human ACTH‐secreting pituitary adenomas

**Figure 5:**
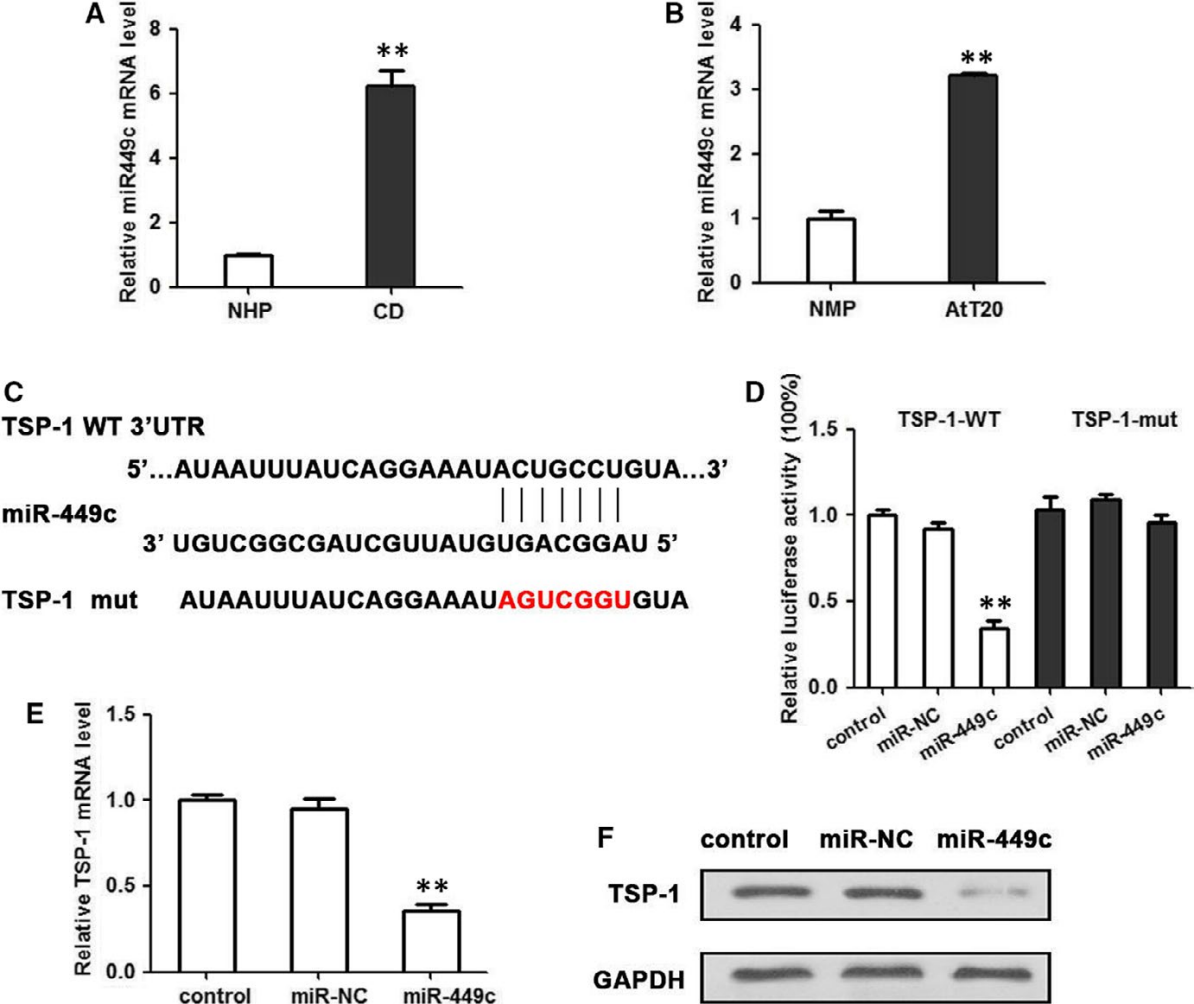
miR‐449c is expressed in pituitary corticotroph tumours and targets the 3′UTR of TSP‐1. A, The expression of miR‐449c mRNA in Cushing's disease (n = 33) and normal pituitary tissue (n = 7) were determined by qRT‐PCR. **P < 0.01. B, The expression of miR‐449c mRNA in AtT20 cells and normal mouse pituitary, tested by qRT‐PCR. C, The predicted target sequence of miR‐449c in the 3′UTR of TSP‐1 and mutant containing three altered nucleotides in the seed sequence of miR‐449c. D, Luciferase assay of pGL3‐TSP‐1WT or pGL3‐TSP‐1 mutant in the presence of miR‐449c mimic in the AtT20 cell line. Luciferase activity was detected 48 h after transfection and normalized to Renilla. (E) qRT‐PCR and (F) Western blotting analysis of miR‐449c effects on mRNA level of TSP‐1 in AtT20 cells. **P < 0.01

**Figure 6:**
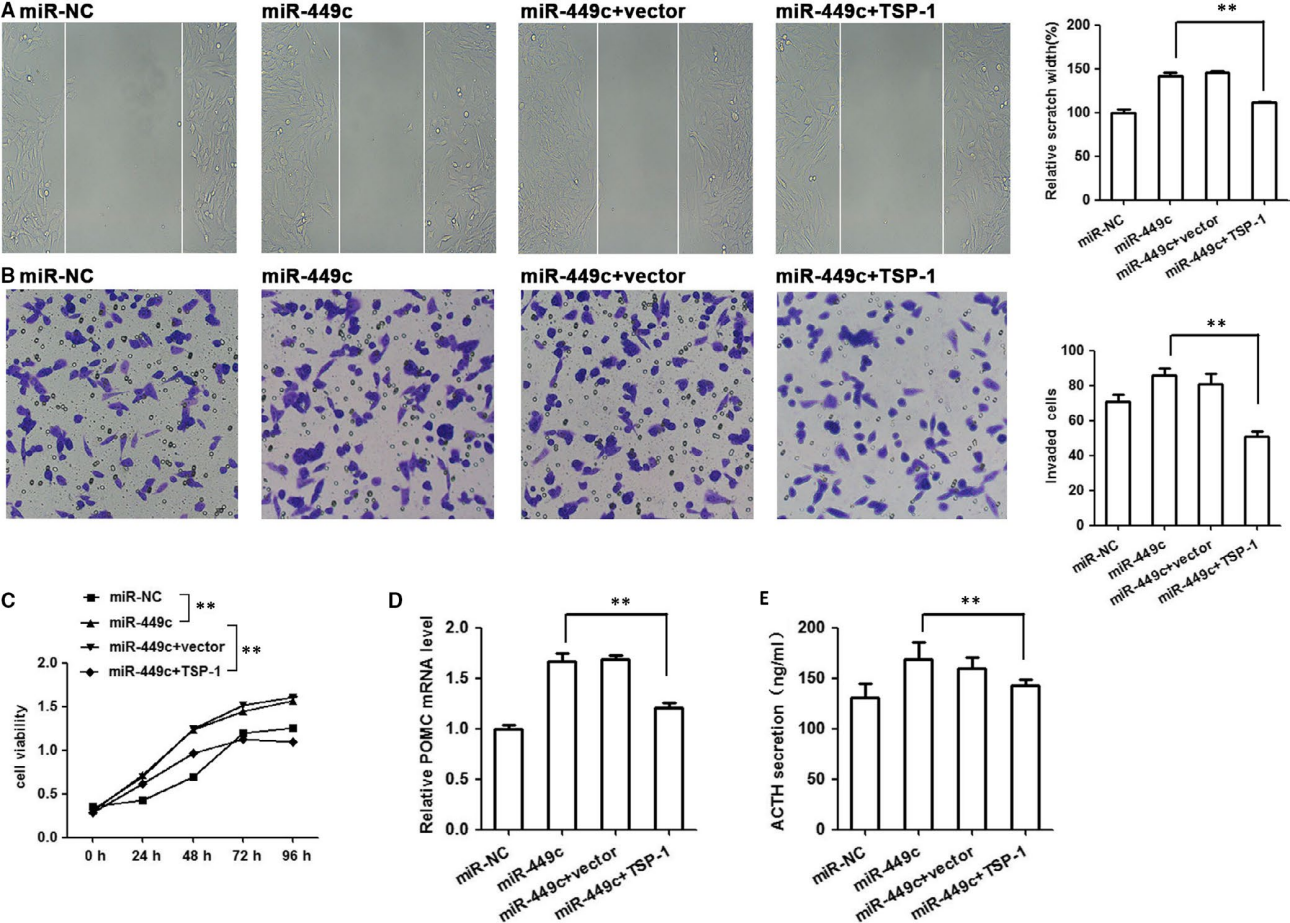
TSP‐1 reversed miR‐449c expression to affect the function of AtT20 cells in vitro. AtT20 cells were transfected with mimics control or miR‐449c and miR‐449c+TSP1. A, Invasion of AtT20 cells in different groups by wound healing assay. B, Transwell invasion assay, (C) MTT assay. (D) qRT‐PCR analysis of miR‐449c and TSP‐1 effects on POMC expression, and (E) miR‐449c and TSP‐1 effects on ACTH secretion. **P < 0.01

The influence of miR‐449c expression on the regulation of TSP‐1 was next examined in pituitary corticotroph tumours. The level of miR‐449c mRNA was found to be significantly elevated in tissue from patients with Cushing's disease (n = 33) compared with normal pituitary tissue (n = 7) (*P* < 0.01) (Figure 5A). The results were found in AtT20 cells (*P* < 0.01 *vs*. normal mouse pituitary) (Figure 5B). The predicted target sequence of miR‐449c in the 3′UTR of TSP‐1 was mutated and interactions were assessed by a Luciferase assay, which confirmed the binding of miR‐449c to the target sequence in 3′UTR of TSP‐1 (Figure 5C,D). qRT‐PCR and Western blotting analysis were then used to assess the effects of overexpressing miR‐449c on the mRNA level of TSP‐1 in AtT20 cells (Figure 5E,F). The presence of miR‐449c significantly reduced the expression of TSP‐1 mRNA (*P* < 0.01) and the detectable levels of protein. However, overexpression of TSP‐1 reversed the effects of miR‐449c in AtT20 cells (Figure 6A‐E). Cell viability, migration and invasion were all increased by the overexpression of miR‐449c but decreased when TSP‐1 was overexpressed. POMC expression and ACTH secretion were also increased in AtT20 cells transfected with miR‐449c but decreased when TSP‐1 was overexpressed in the cells. Taken together, these results imply that TSP‐1 expression inversely correlates with miR‐449c expression in human ACTH‐secreting pituitary adenomas.

### 3.4 lncTHBS1 and TSP‐1 correlate through targeting by miR‐449c and a reciprocal negative regulation existed between miR‐449c and lncTHBS1

**Figure 7:**
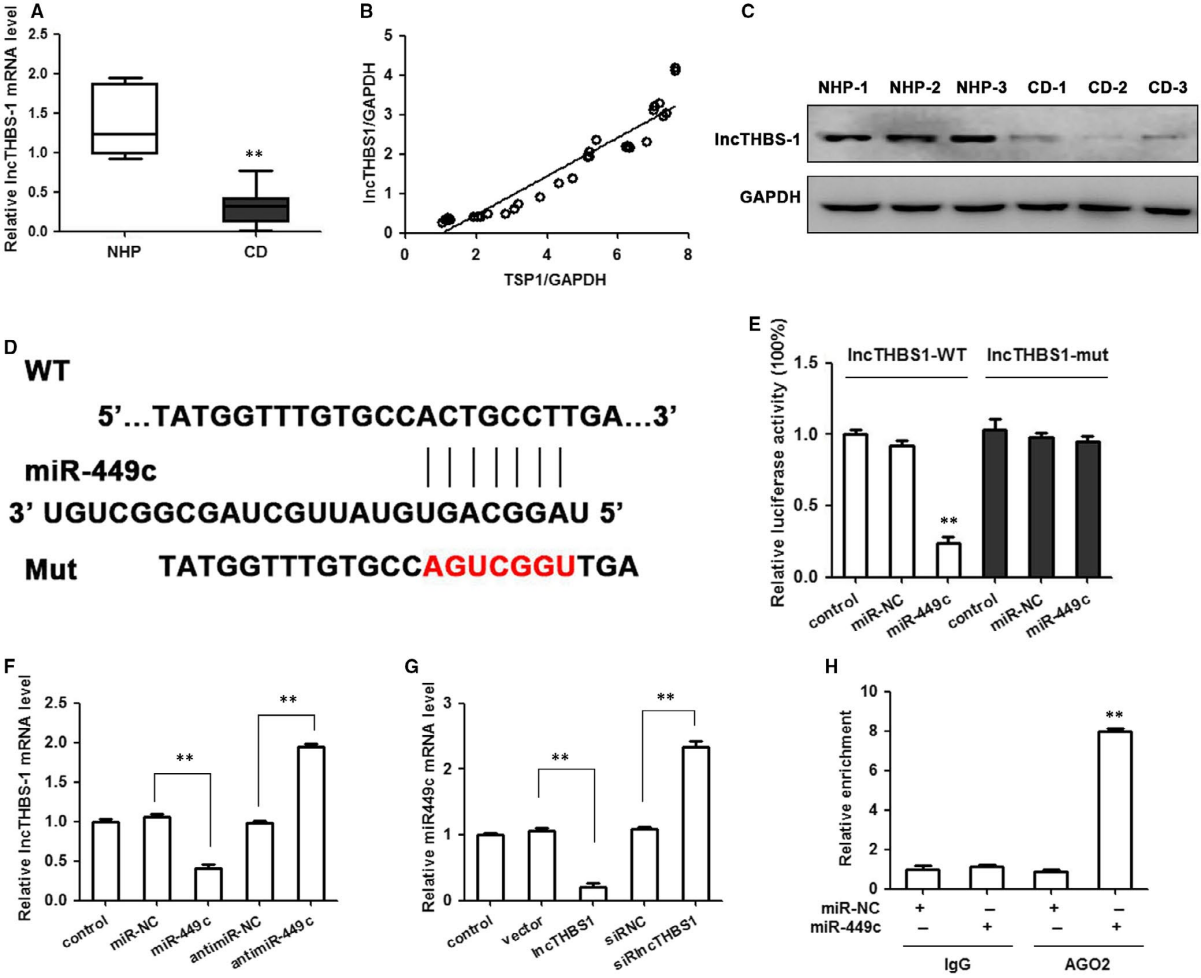
Positive correlation between TSP‐1 and lncTHBS1 expression. lncTHBS1 targets miR‐449c by directly binding to a miRNA response element. A, The expression of lncTHBS1 mRNA in CD (n = 33) and normal pituitary tissue (n = 7). B, Correlation analysis of the relationship between lncTHBS1 expression and TSP‐1 level. C, Northern blot assays were used to detect lncTHBS1 expression in CD. D, Schematic representation of the predicted binding sites for miR‐449c, and the site mutagenesis design for the reporter assay. E, The relative luciferase activities were inhibited in the HEK‐293T cells transfected with the reporter vector lncTHBS1‐WT, but not with the reporter vector lncTHBS1‐Mut. F, miR‐449c expression decreased lncTHBS1 expression, and inhibition of miR‐449c enhanced lncTHBS1 expression in the HEK‐293T cells. G, lncTHBS1 expression decreased miR‐449c expression whereas inhibition of lncTHBS1 increased miR‐449c expression in HEK‐293T cells. H, miR‐449c was identified in the lncTHBS1 complex. miR‐NC and miR‐449c cell lysates were used for RNA‐IP with anti‐Ago2 antibody. Cells transfected with miR‐449c mimics or miR‐NC, followed by qRT‐PCR to detect lncTHBS1. **P < 0.01

On the basis of bioinformatics website prediction and in connection with previous findings from whole transcriptome analysis of adenoma tissue from patients with Cushing's disease and normal pituitary tissue,^18^ proposed that the aberrantly expressed lncTHBS1 may play an important role for the regulation of TSP‐1 in Cushing's disease. Firstly, we confirmed that the expression of lncTHBS1 mRNA was lower in pituitary ACTH‐secreting (n = 33) adenoma compared with normal pituitary tissue (n = 7) (Figure 7A). Furthermore, the correlation analysis confirmed a relationship between lncTHBS1 and TSP‐1 expression (Figure 7B). Northern blot analysis detected a low level of lncTHBS1 expression in pituitary corticotroph adenoma compared with normal pituitary tissue (Figure 7C). The predicted miRNA response element binding site for miR‐449c was mutated in lncTHBS1 and confirmed by Luciferase activity in HEK‐293T cells (Figure 7D,E). MiR‐449c expression decreased lncTHBS1 expression and inhibition of miR‐449c enhanced lncTHBS1 expression in HEK‐293T cells (Figure 6F). Whereas, lncTHBS1 expression decreased miR‐449c expression while inhibition of lncTHBS1 increased miR‐449c expression (Figure 6G). Moreover, miR‐449c was identified in an lncTHBS1 complex using RNA‐IP with anti‐Ago2 antibody (Figure 6H). These results indicate a positive correlation between TSP‐1 and lncTHBS1 expression and demonstrate that lncTHBS1 targets miR‐449c by directly binding to a miRNA response element.

## 4 DISCUSSION

ACTH‐secreting pituitary adenomas, originating from pituitary corticotroph cells, are related to substantial morbidity and cause adrenal hypercortisolaemia, which is often referred to as Cushing's disease.^47^ The pathogenesis of Cushing's disease is obscure and it remains difficult to treat successfully.^48^, ^49^, ^50^ The present study aimed to elucidate the mechanistic role of TSP‐1 in regulating the tumour function of pituitary adenomas. We demonstrated that TSP‐1 was significantly lower in Cushing's disease than in normal pituitary tissue and that forced overexpression of TSP‐1 in AtT20 cells decreased POMC transcription and ACTH secretion. Moreover, we found that the overexpression of TSP‐1 decreased POMC transcription and ACTH secretion in AtT20 cells and significantly decreased cell viability, colony formation and increased the ratio of apoptosis and suppressed migration and invasion. TSP‐1 overexpression suppressed tumour growth in vivo too. TSP‐1 is a direct target of miR‐449c and miR‐449c activity enhanced tumorigenesis by directly inhibiting TSP‐1 expression. POMC expression and ACTH secretion were also increased in AtT20 cells transfected with miR‐449c but decreased when TSP‐1 was overexpressed.

In the present study, we also assessed protein levels of Ki67, VEGF and MMP9 in relation to TSP‐1 expression. The presence of Ki67 was more intense in the nuclei of tissue samples from patients with Cushing's disease, which indicated increased cell proliferation. In previous studies, the expression of Ki67, VEGF and MMP9 appear to be elevated in Cushing's disease.^38^, ^40^ Ki‐67 is a marker of cell proliferation and is found to occur at significantly higher levels in invasive compared with non‐invasive adenomas.^51^ The growth factor VEGF can induce endothelial cell proliferation and the permeabilization of blood vessels, promote cell migration, inhibit apoptosis and is actively involved in the angiogenesis, vasculogenesis and endothelial cell growth of several cancers including colorectal cancer^52^ and ovarian granulosa cell tumour.^53^ In pituitary adenomas, VEGF expression is well preserved and might contribute to the vascular supply of tumours.^54^. The inhibitory effect of TSP‐1 on VEGF‐mediated angiogenesis is also proposed to involve the TSP‐1 receptor CD36 and endothelial cell apoptosis pathways.^55^ A recent study evaluating MMP9, the pituitary tumour transforming gene (PTTG), high mobility group A 2 (HMGA2) and Ki‐67 in recurrent and non‐recurrent ACTH‐secreting pituitary tumours, has found that high levels of MMP9 are associated with a greater reoccurrence and a shorter recurrence‐free interval.^38^ The same study found that PTTG, HMGA2 and Ki‐67 expression were not significantly different between recurrent and non‐recurrent ACTH‐secreting pituitary tumours. In the present study, VEGF and MMP9 appeared to be upregulated and located in the cytoplasm whereas Ki67 was upregulated and located in the nucleus. The presence of TSP‐1 was less obvious in the pituitary tissue of patients with Cushing's disease, confirming that TSP‐1 is downregulated in Cushing's disease whereas Ki67, MMP9 and VEGF are upregulated. However, when TSP‐1 is overexpressed MMP9 and matrix metalloproteinases MMP2 and MMP7 are downregulated.

LncRNAs have been implicated in various cellular functions including chromatin remodelling, genomic imprinting, nuclear compartmentalization, splicing, cell cycle progression and cellular reprogramming.^56^, ^57^, ^58^ A larger number of studies have found that the aberrant expression of lncRNAs played a considerable roles in human tumorigenesis, and numerous lncRNAs have been documented in various cancers throughout the body, including the brain, breast, liver and pancreas.^57^, ^59^, ^60^, ^61^ Mechanistically, aberrant lncRNAs were associated with a range of interaction partners, including transcription factors, RNA binding proteins, nascent RNA transcripts, DNA, chromatin and microRNA.^62^ In this study we confirmed a correlation analysis between lncTHBS1 and TSP‐1 expression and that miR‐449c expression decreased lncTHBS1 expression, whereas, lncTHBS1 expression decreased miR‐449c expression. In addition, miR‐449c was identified in an lncTHBS1 complex using RNA‐IP with anti‐Ago2 antibody. RNA‐IP is a novel strategy that exploits the involvement of Argonaute proteins in the translational repression complex associated with microRNA.^63^ The technique involves the immunoprecipitation of whole miR‐silencing complexes containing miRs and associated target mRNAs.^64^ Our results indicate a positive correlation between TSP‐1 and lncTHBS1 expression and demonstrate that lncTHBS1 targets miR‐449c by directly binding to a miRNA response element. Therefore, lncTHBS1 may function as a competitive endogenous RNA with miR‐449c.

In conclusion, our present work reveals that TSP1 expression is downregulated in Cushing's disease. Forced overexpression of TSP‐1 suppressed proliferation, migration and invasion in pituitary adenoma cells and decreased POMC transcription and ACTH secretion. Moreover, we found that TSP‐1 is a direct target of miR‐449c and that miR‐449c activity enhanced tumorigenesis by directly inhibiting TSP‐1 expression, this suggests that TSP‐1 might be a negative regulator of miR‐449c. lncTHBS1 and TSP‐1 correlated through interactions with miR‐449c and a reciprocal negative regulation existed between miR‐449c and lncTHBS1. These results suggest that lncTHBS1 might function as ceRNA for miR‐449c, which could suppress the development of Cushing's disease. Our data shed new light on the understanding of the interaction between lncRNA and miRNA in the molecular regulation of human pituitary corticotroph tumours.

## CONFLICT OF INTEREST

The authors confirm that there are no conflicts of interest.
